# Spatiotemporal Dynamics of Ammonia-Oxidizing Thaumarchaeota in Distinct Arctic Water Masses

**DOI:** 10.3389/fmicb.2018.00024

**Published:** 2018-01-23

**Authors:** Oliver Müller, Bryan Wilson, Maria L. Paulsen, Agnieszka Rumińska, Hilde R. Armo, Gunnar Bratbak, Lise Øvreås

**Affiliations:** ^1^Department of Microbiology, University of Bergen, Bergen, Norway; ^2^University Center in Svalbard (UNIS), Longyearbyen, Norway

**Keywords:** thaumarchaeota, ammonia-oxidation, Arctic Ocean, water mass, ecotype, *amoA*, 16S rRNA gene sequencing

## Abstract

One of the most abundant archaeal groups on Earth is the Thaumarchaeota. They are recognized as major contributors to marine ammonia oxidation, a crucial step in the biogeochemical cycling of nitrogen. Their universal success is attributed to a high genomic flexibility and niche adaptability. Based on differences in the gene coding for ammonia monooxygenase subunit A (amoA), two different ecotypes with distinct distribution patterns in the water column have been identified. We used high-throughput sequencing of 16S rRNA genes combined with archaeal *amoA* functional gene clone libraries to investigate which environmental factors are driving the distribution of Thaumarchaeota ecotypes in the Atlantic gateway to the Arctic Ocean through an annual cycle in 2014. We observed the characteristic vertical pattern of Thaumarchaeota abundance with high values in the mesopelagic (>200 m) water throughout the entire year, but also in the epipelagic (<200 m) water during the dark winter months (January, March and November). The Thaumarchaeota community was dominated by three OTUs which on average comprised 76% ± 11 and varied in relative abundance according to water mass characteristics and not to depth or ammonium concentration, as suggested in previous studies. The ratios of the abundance of the different OTU types were similar to that of the functional *amoA* water cluster types. Together, this suggests a strong selection of ecotypes within different water masses, supporting the general idea of water mass characteristics as an important factor in defining microbial community structure. If indeed, as suggested in this study, Thaumarchaeota population dynamics are controlled by a set of factors, described here as water mass characteristics and not just depth alone, then changes in water mass flow will inevitably affect the distribution of the different ecotypes.

## Introduction

The discovery of the high abundance of marine planktonic Archaea in 1992 was a revelation (DeLong, 1992; Fuhrman et al., 1992). Since then, numerous studies have confirmed both their high proportions and population dynamics, especially in deeper waters and from both polar oceans (Massana et al., 1998; Murray et al., 1998). In later studies the marine Archaea have been found to play important roles in many biogeochemical processes (Ouverney and Fuhrman, 2000; Offre et al., 2013). When Craig Venter and colleagues discovered genes encoding for ammonia monooxygenase subunit A (amoA) in their metagenome analyses from the Sargasso Sea, new information regarding these processes was provided, leading to a particular interest in the marine Thaumarchaeota (Venter et al., 2004). This interest was further strengthened with the cultivation and characterization of the first marine archaeal isolate (*Candidatus Nitrosopumilus maritimus* SCM1) capable of ammonia oxidation (Könneke et al., 2005). Today, chemoautotrophic ammonia oxidizing Archaea (AOA) are recognized as the major contributors to marine microbial ammonia oxidation and thus driving nitrification processes, dominating these relative to their bacterial ammonia oxidizing (AOB) counterparts (Wuchter et al., 2006; Valentine, 2007).

Thaumarchaeota are widely distributed and may make up a significant part of marine microbial communities (Karner et al., 2001; Agogué et al., 2008; Beman et al., 2008). In the surface waters of polar regions there seem to be temporal changes in the relative abundance of Thaumarchaeota with an increase during winter and decline in summer (Massana et al., 1998; Murray et al., 1998; Church et al., 2003; Alonso-Sáez et al., 2008; Grzymski et al., 2012). Photoinhibition of ammonia oxidation has been hypothesized as an underlying cause for the seasonal disappearance of AOA (Guerrero and Jones, 1996; Murray et al., 1998; Mincer et al., 2007; Merbt et al., 2012). However, other factors, such as competition with an increasing abundance of phytoplankton and associated bacterial blooms (Massana et al., 1998; Church et al., 2003; Herfort et al., 2007) or nutrient limitations, including ammonium (Wuchter et al., 2006; Herfort et al., 2007; Kirchman et al., 2007), may also play important roles. Physical aspects such as deep water mixing have been suggested to resolve the winter increase of Thaumarchaeota abundance in the Southern Oceans (Kalanetra et al., 2009; Grzymski et al., 2012), but this could not explain the same trends in the Arctic, where the ocean remains relatively stratified during winter (Forest et al., 2011). Recent data have suggested that the increase in AOA is due to *in situ* growth at the surface and not to mixing with deeper water masses (Alonso-Sáez et al., 2012).

The surface Thaumarchaeota populations comprise predominantly one type of AOA, while the deep ocean is dominated by another type of AOA and have thus far, based on differences in their *amoA* genes, been divided into a surface (WCA) and a deep (WCB) type (Francis et al., 2005; Hallam et al., 2006; Beman et al., 2008; Sintes et al., 2013). Their depth-dependent distribution has been demonstrated in many different regions, including the Gulf of California (Beman et al., 2008), the Gulf of Mexico (Tolar et al., 2013), the Arctic Ocean (Pedneault et al., 2014), Monterey Bay (Smith et al., 2014) and throughout the entire Atlantic Ocean (Sintes et al., 2016). Taxonomically, *amoA* sequences can be divided into six main subclusters all branching to the *N. maritimus* cluster (Pester et al., 2012; Sintes et al., 2016). Two subclusters include only WCA sequences and the other four subclusters include exclusively WCB sequences.

The abundance of the different AOA types has also been correlated with ammonium concentrations and this has led to the introduction of high and low ammonium concentration AOA (HAC-AOA and LAC-AOA, respectively) (Herfort et al., 2007; Kirchman et al., 2007; Sintes et al., 2013). HAC-AOA dominate at depths with high ammonium concentrations while LAC-AOA are in higher abundance in deeper ocean regions where the ammonium concentration is low (Sintes et al., 2013). Overall, LAC-AOA corresponded taxonomically to WCB-types and HAC-AOA with WCA types. Ammonium concentrations (Woodward and Rees, 2001; Varela et al., 2007; Clark et al., 2008) measured for different oceanic regions could also support an observed macroecological AOA distribution in the Atlantic Ocean (Sintes et al., 2016). However, other environmental factors such as depth, temperature, dissolved oxygen, nitrite, and salinity have been previously identified as influences on the abundance and diversity of AOA (Francis et al., 2005; Herfort et al., 2007; Abell et al., 2010; Santoro et al., 2010; Biller et al., 2012; Pester et al., 2012; Sintes et al., 2015). Overall, the niche specification of the two ecotypes is best explained by depth in combination with geographic region and to a lesser extent with environmental factors, including ammonium concentration. However, it remains unclear whether these taxonomic definitions, both WCA/WCB and HAC/LAC, can be used to associate observed abundances with distinct biogeochemical niches, like water masses.

We have recently reported the high relative abundance and seasonal variation of Thaumarchaeota in waters around the western coast of Svalbard (Wilson et al., 2017). Here we extend the studies and investigate the Thaumarchaeota community in five different water masses, dominated by Atlantic and Arctic Water, using high throughput 16S rRNA gene sequencing aiming to identify different Thaumarchaeota populations and using *amoA* gene abundance to elucidate functional capabilities that may influence their distribution and dynamics.

## Materials and methods

### Study site and sampling

**Figure 1:**
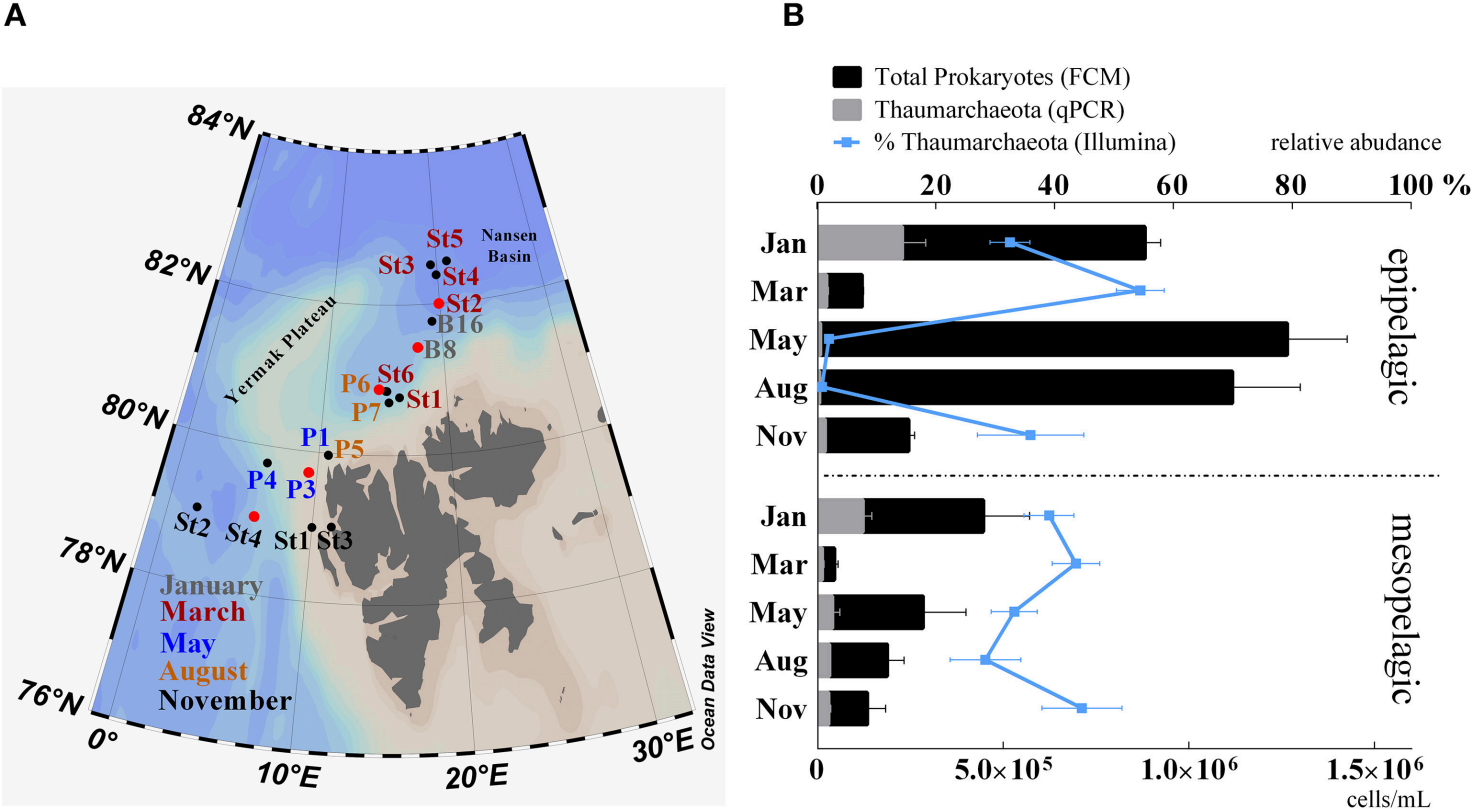
**(A)** Study area Northwest of Svalbard. Stations are colored according to their time of sampling: gray, January; red, March; blue, May; yellow, August; black, November. Stations marked with a red circle were selected for *amoA* functional gene analyses. **(B)** Abundance of prokaryotes counted by flow cytometry (black bars) and superimposed abundance of Thaumarchaeota (gray bars) determined by qPCR of 16S rRNA genes. Relative abundance of Thaumarchaeota in relation to the total of 16S rRNA genes from Illumina sequencing data is shown by the blue line. Samples were grouped in epipelagic (0–200 m; *n* = 28) and mesopelagic (200–1,000 m; *n* = 22) zones and according to their sampling month. Standard errors were calculated for average values and are indicated as error bars.

Samples were collected as part of the MicroPolar project (in cooperation with the project “CarbonBridge”) during five cruises in 2014 north-west of Svalbard, following several transects along the West Spitsbergen Current (WSC) at the eastern part of the Fram Strait up to the Arctic Ocean (Figure 1). This area is hydrographically characterized by three Atlantic water masses, including Atlantic Water (AW), cold Atlantic Water (cAW) and Intermediate Water (IW), having salinity >34.9 and temperatures >2°C, 0–2°C and <0°C, respectively; and also, by two Arctic water masses, Surface Water (SW) and Arctic Water (ArW), having salinity < 34.92 and density (σ_t_) <27.7 and >27.7 respectively (Cokelet et al., 2008; de Steur et al., 2014; Randelhoff et al., 2015). An overview of the water mass characteristics is listed in Supplementary Table S3. The WSC at the eastern part of the Fram Strait transports Atlantic water into the Arctic Ocean. This Atlantic water can also be found in deeper mesopelagic zones as cAW and IW. The water masses classified as Arctic Water do not necessarily originate from the Arctic Ocean interior, but have undergone similar freshening and cooling processes and have the same physical characteristics as Arctic Ocean water masses.

Sampling periods extended over an entire polar year with cruises in January (06.01–15.01), March (05.03–10.03), May (15.05–02.06), August (07.08–18.08), and November (03.11–10.11). Depth profiles of temperature, salinity and fluorescence were recorded using a SBE 911plus CTD system (Sea-Bird Scientific, WA, USA) and used to identify water masses and to collect water for downstream analyses. Samples (25-50 L) for molecular analyses were taken between depths of 1 and 1,000 m (Supplementary Table S1), filtered onto 0.22 μm pore size Millipore® Sterivex filters (Merck-Millipore, MA, USA) and immediately frozen at −80°C. In total 50 samples (epipelagic zone; 0–200 m; *n* = 28 and mesopelagic zone; 200–1,000 m; *n* = 22) were used for molecular analysis. Further cruise and sampling details are described in Paulsen et al. (2016) and Wilson et al. (2017), respectively.

### Flow cytometry

The abundance of prokaryotes was detected from samples collected at 18 stations from 11 depths (1, 5, 10, 20, 30, 50, 100, 200, 500, 750, and 1,000 m) during 5 cruises using an Attune® Acoustic Focusing Flow Cytometer (Applied Biosystems by Life technologies, CA, USA) with a syringe-based fluidic system and a 20 mW 488 nm (blue) laser. First, samples were fixed with glutaraldehyde (0.5% final conc.) and incubated at 4°C for a minimum of 30 min, frozen in liquid nitrogen and stored at −80°C. For analysis, samples were diluted with 0.2 μm filtered TE buffer (Tris 10 mM, EDTA 1 mM, pH 8), stained with a green fluorescent nucleic acid dye (SYBR Green I; Molecular Probes, Eugene, Oregon, USA) and kept for 10 min at 80°C in a water bath (Marie et al., 1999). A minimum of 100 μL was counted at a low flow rate of 25 μL min^−1^ and prokaryotes were discriminated on a biparametric plot of green florescence vs. red florescence.

### Ammonium measurements

Concentrations of ${\text{NH}}_{4}^{+}$ were determined fluorometrically from frozen samples (4 mL) using orthophthadialdehyde according to the protocol by Holmes (1999). The method was adapted for microplate readings following (Poulin and Pelletier, 2007) and samples were analyzed on a 2300 EnSpire™ Multilabel Plate Reader (PerkinElmer, Finland). A 0.1 M ammonium chloride stock solution was used to prepare standard curves (0.1, 0.3, 0.6, 1, 2 μM) with correlation coefficients ≥0.986.

### Nucleic acids extraction and amplification for amplicon sequencing

DNA and RNA from Sterivex filters were extracted using the AllPrep DNA/RNA Mini Kit (Qiagen, Hilden, Germany). Details regarding RNA processing can be found in Wilson et al. (2017). In short, 10 ng RNA was treated with the DNA-free DNA Removal kit (Invitrogen, CA, USA) and subsequently reverse transcribed using the SuperScript III First-Strand Synthesis System for RT-PCR (Invitrogen), following the manufacturer's instructions. DNA was amplified using a two-step nested PCR approach with primers 519F and 806R (Supplementary Table S2) targeting both the archaeal and the bacterial 16S rRNA gene V4 hypervariable region. During the first step, triplicate samples were amplified in reaction volumes of 20 μL, comprising 10 ng DNA, 10 μL HotStarTaq Master Mix (Qiagen), 0.5 μM of each primer and nuclease-free water. PCR reaction conditions were as follows: initial denaturation of 15 min at 95°C, followed by 25 cycles of 95°C for 20 s, 55°C for 30 s and 72°C for 30 s and a final extension step of 72°C for 7 min. Triplicate PCR products were pooled and purified using the DNA Clean & Concentrator-5 kit (Zymo Research Corporation, CA, USA). 10 ng of pooled PCR product was used for the second PCR step, in a reaction volume of 50 μL together with 25 μL HotStarTaq Master Mix, 0.5 μM of each nested primer (containing a unique eight-nucleotide barcode) and nuclease-free water. PCR reaction conditions were as follows: initial denaturation of 15 min at 95°C, followed by 15 cycles of 95°C for 20 s, 62°C for 30 s, 72°C for 30 s and a final extension step of 72°C for 7 min. Final PCR products were purified using Agencourt AMPure XP Beads (Beckman Coulter Inc., CA, USA) and prepared for sequencing by pooling the samples in equimolar amounts. The quality and concentration of the amplicon pool were assessed by agarose gel electrophoresis and a Qubit 3.0 Fluorometer, respectively. Libraries were sequenced at the Norwegian Sequencing Centre (Oslo, Norway) using their Illumina MiSeq platform (MiSeq Reagent Kit v2, Illumina, CA, USA). Sequencing data are available at the European Nucleotide Archive (ENA) under study accession number PRJEB23129. The primers (519F-806R) used in this study have been shown to have a low affinity for the SAR11 cluster, which can result in overestimation of other prokaryotic groups (Apprill et al., 2015).

### 16s rRNA gene sequence analysis

Paired-end sequences were processed using various bioinformatic tools incorporated in the QIIME software environment (Caporaso et al., 2011), as described in Paulsen et al. (2016). Briefly, FASTQ files were quality end-trimmed, merged and prokaryotic OTUs were selected at a sequence similarity threshold of 97% and taxonomy assigned using the Silva 111 reference database (Quast et al., 2013). A total of 5,995,334 sequences were retrieved from high-throughput sequencing of the 16S rRNA gene V4 hypervariable region from DNA across fifty samples from five cruises. After removal of singletons, unassigned OTUs and chloroplast reads, sequences were rarefied to 10,000 reads per sample, with a total of 24,723 unique OTUs (63.1% singletons) at 97% sequence similarity. Bray–Curtis resemblance and ANOSIM statistical analysis were performed using PRIMER-E (Version 6; Quest Research Limited, Auckland, NZ).

### Quantitative real-time PCR (qPCR)

All qPCR assays were run in triplicates on a C1000 Thermocycler (BioRad, CA, USA). The following qPCR reaction mixture was used: 10 μl Fast EvaGreen® qPCR Master Mix (Biotium, Inc., Hayward, CA, USA), 0.5 μM final concentration of each primer, 1 μL template DNA (corresponding to 1 ng of environmental DNA) and water were added to a final volume of 20 μL. All qPCR reactions were performed in white 96 well plates (BioRad). Thaumarchaeota 16S rRNA genes were quantified using the Thaumarchaeota specific forward primer Thaum-494F (Hong et al., 2015) and an archaeal universal primer ARC917R (Loy et al., 2002). This primer pair was suggested to better target the Thaumarchaeota and showed a higher affinity (96%) *in silico* to Marine Group I Archaea than previously used primer pairs (Hong et al., 2015). qPCR reaction conditions were as follows: initial activation for 2 min at 95°C, followed by 35 cycles of amplification, including denaturation at 95°C for 30 s, annealing at 55°C for 30 s, extension at 72°C for 30 s and a final extension step of 10 min at 72°C. The fluorescence was measured at the end of each cycle and a melting curve obtained from 65 to 95°C, with increments of 0.2°C. Ten-fold dilutions ranging from 1.1 × 10^8^ to 1.1 × 10^3^ copies of environmental Thaumarchaeota 16S rRNA gene were used as a quantification standard. Efficiencies for all qPCR reactions ranged from 83 to 84% with constant *R*^2^-values of 0.998. To calculate gene copies per mL, the copy number per ng was multiplied by the DNA concentration per mL (based on flow cytometer counts and the assumption that one prokaryote contains 3 fg of DNA; Fuhrman and Azam, 1982; Jeffrey et al., 1996).

### Phylogenetic analysis of amoA and 16s rDNA clone libraries

A total of 10 MicroPolar samples from five stations representative of all five water masses were selected for *amoA* functional gene amplification. Each time point comprised both a surface and deep sample, excluding surface samples from the summer season (May and August), due to very low *amoA* abundances. Overall, eight DNA and two RNA samples were used for this analysis. The two RNA samples were from the same depths as the DNA samples from the November cruise and are included as an indicator of the active transcription of *amoA* mRNA. Amplification was performed using archaeal *amoA* primers (Supplementary Table S2) targeting a 635 bp gene fragment using the protocol of Francis et al. (2005) and 30 amplification cycles (iCycler, Bio-Rad, CA, USA). These primers have been widely used, but have been shown to underestimate *amoA* abundance in surface water samples (Tolar et al., 2013). PCR products were purified using the ExoSap-IT kit (Applied Biosystems) and subsequently cloned with the Qiagen PCR Cloning Kit (Qiagen) following manufacturer instructions. A total of 242 clones from all 10 samples were selected, and sequenced in-house at the sequencing facility of the University of Bergen (http://www.uib.no/en/seqlab). In order to obtain the 16S rRNA gene fragments for phylogenetic analysis, the same steps were followed as for the *amoA* genes; amplification was performed using an Archaea-specific forward primer in combination with a universal prokaryotic reverse primer resulting in amplicons of 1481 bp length (Supplementary Table S2). PCR reaction conditions were similar to those described before, with the exception of the annealing temperature, which was adjusted to 52°C. All *amoA* gene sequences from this study have been deposited at ENA under study accession number PRJEB23151. The three full length sequences of the 16S rRNA gene have been deposited at NCBI under GenBank accession numbers MG238502- MG238504.

Sequencing of *amoA* clones resulted in a total of 230 high quality sequences. This dataset was combined with an additional 254 *amoA* sequences (220 bp gene fragment) from a recent study on archaeal ammonia oxidizing ecotypes in the Atlantic Ocean (Sintes et al., 2016). This combined dataset was used to define OTUs at 97% sequence similarity using the *de novo* uclust (Edgar, 2010) OTU clustering method in QIIME, using default parameters. In total, 189 OTUs were identified and used for phylogenetic analysis based on multiple alignments of *amoA* OTUs using MUSCLE (Edgar, 2004) with default parameters. The phylogenetic tree was inferred using the neighbor–joining method (Saitou and Nei, 1987) with 1000 bootstrap replicates. The retrieved tree was viewed using Evolview v2 (He et al., 2016).

The same strategy was implemented for Thaumarchaeota16S rRNA gene sequences. In order to include the Illumina amplicon reads, all sequences used (clonal or otherwise) for the phylogenetic analysis were trimmed to a size of 268 bp. A total of 1256 Thaumarchaeota sequences, including the three most abundant Thaumarchaeota OTUs from our amplicon data set, three full length 16S rRNA Sanger-sequenced reads and 1242 environmental sequences from the Arctic Ocean, the Atlantic Ocean, the Northeast Pacific, the North Sea and Gulf of Mexico were used to define OTUs at 97% similarity (Agogué et al., 2008; Bale et al., 2013; Tolar et al., 2013; Wright, 2013; Ijichi and Hamasaki unpublished). The resulting 23 OTUs were used for phylogenetic analysis as described above. We calculated the relative abundance of these OTUs in sets of samples from depths below or above 100 m.

## Results

### Hydrography and seasonal thaumarchaeota abundance

We used 16S rRNA gene sequencing and qPCR analyses to determine the relative and absolute abundance of Thaumarchaeota in samples taken during five cruises throughout the year in the Arctic Ocean off the western coast of Svalbard (Figure 1). Throughout the sampling period, Thaumarchaeota represented up to 73% (62% by qPCR) of the prokaryotic community. The relative abundance of Thaumarchaeota varied with both depth and season. Surface samples from the epipelagic (1–200 m) zone showed clear seasonal changes in Thaumarchaeota relative abundance. During the winter months relative abundance was high (44% ±15), while it was low during the summer season (1.4% ±1.4). In contrast, Thaumarchaeota relative abundance in the deep mesopelagic samples (200–1,000 m) was relatively high (38% ±11) throughout the entire year (Figure 1B).

**Figure 2:**
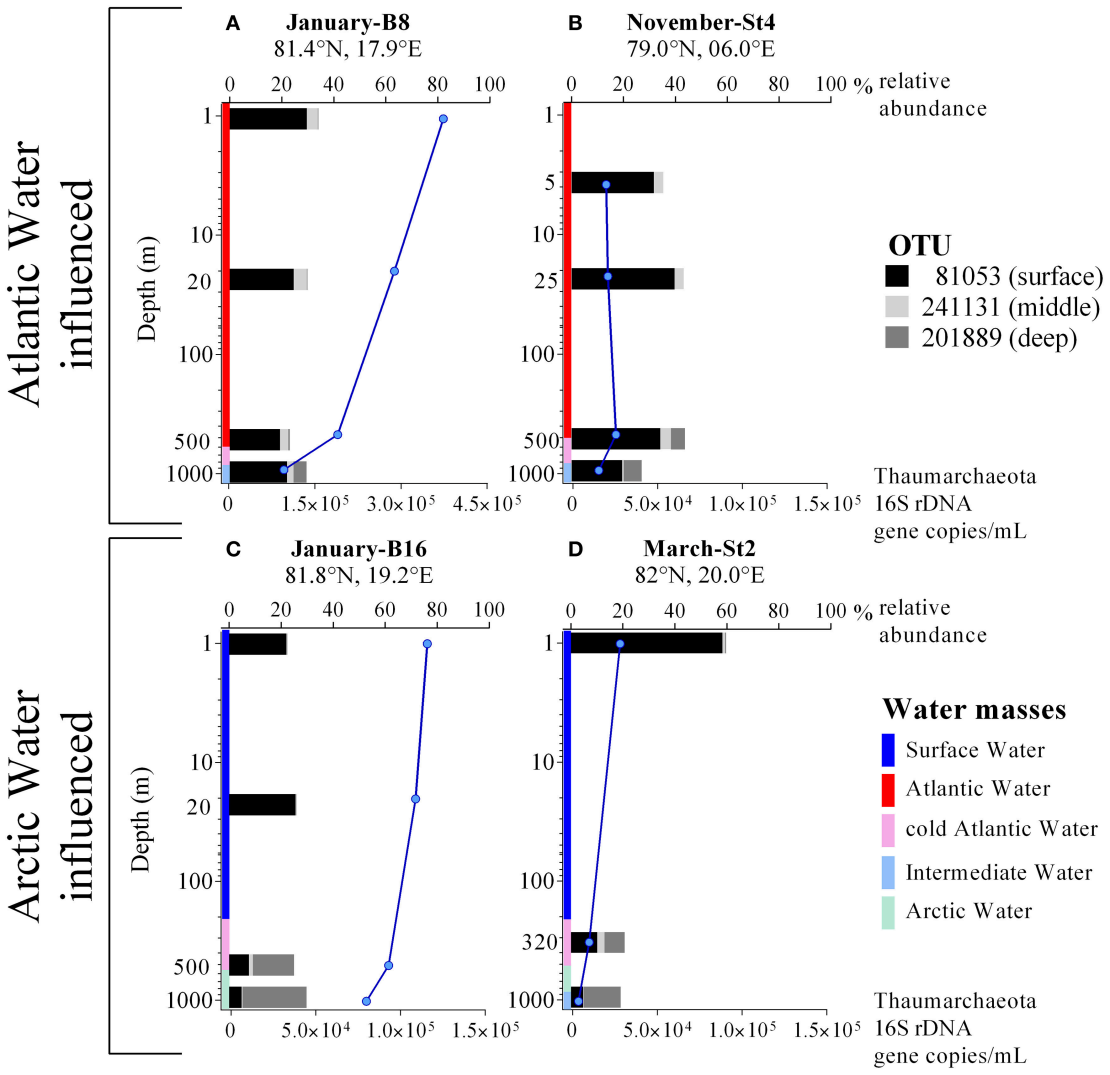
**(A–D)** Profiles of Thaumarchaeota abundance (blue lines) determined by qPCR of 16S rRNA genes and Thaumarchaeota relative abundance (black, gray, light gray bars; in % of total 16S rRNA Illumina amplicons) from four different stations, where either Atlantic or Arctic water masses were dominating. Notice different scales on the x-axis (lower x-axis for qPCR derived Thaumarchaeota gene copies per mL; upper x-axis for Thaumarchaeota relative abundance from Illumina sequencing data). Colors indicate the water mass profile. Note that the y-axis is in logarithmic scale.

Although total prokaryote abundance strongly increased during the summer months, absolute Thaumarchaeota abundance of up to 3.8 × 10^5^ cells mL^−1^ (January, 1 m, station B8) was highest during the winter months (Figures 1B, 2). Relative Thaumarchaeota abundance values from 16S rRNA gene sequencing and calculated values from qPCR were comparable, while the Illumina derived relative abundance was on average 14% higher (Supplementary Figure S3). We identified the three most abundant OTUs, which constituted on average 76% ±11 of the total Thaumarchaeota community in all samples. The same three OTUs were identified from 16S rRNA sequencing of reverse-transcribed total RNA, suggesting an active role in the prokaryotic community (Wilson et al., 2017).

Phylogenetic analysis of 16S rRNA genes, which included the three OTUs, and 1,242 environmental sequences from the Arctic Ocean, the Atlantic Ocean, the Northeast Pacific, the North Sea and Gulf of Mexico, showed that the three most abundant OTUs from our study are also represented in other environments. The OTUs representing our three most abundant Thaumarchaeota types comprise 92% of all sequences included in the analysis. The phylogenetic tree shows that all OTUs are related to the cultured strain *Nitrosopulimus maritimus* SCM1 and that they divide into two subgroups representing predominantly samples of either epipelagic or mesopelagic origin (Supplementary Figure S1). While OTUs affiliated to the surface group can be found in samples from both the epipelagic and mesopelagic zones, OTUs from the deep group were exclusively from mesopelagic samples indicating a depth-dependent distribution pattern as documented before.

### Thaumarchaeota abundance patterns correlated with specific water masses

The profiles in Figures 2A–D illustrate the differences in abundance of Thaumarchaeota OTUs for the five cruises and in contrasting stations with varying water masses. Overall, similar presence/absence patterns of the three most abundant OTUs can be observed throughout all cruises, which are partly connected to depth (Figure 2). This includes OTU 81053 and OTU 201889 being most abundant in the surface and deep waters, respectively. In order to identify a distribution pattern for these OTUs, we separated all samples, according to their depth in epipelagic and mesopelagic groups, as has been done previously (**Figure 4A**). The OTU abundance pattern between the two water zones was significantly different, as shown by an ANOSIM analysis (*R* = 0.32; *p* = 0.001). Whilst OTU 81053 was most abundant in epipelagic waters (62–88%), it was found to be highly variable in samples from the mesopelagic zone (9.8–71%), thus referred to as “surface OTU”. In contrast, OTU 201889 was barely detectable in epipelagic samples (<0.5%) and of varying abundance (0.2–51%) in mesopelagic samples, hence was referred to as “deep OTU”. The third most abundant OTU (0.2–19%) was detected in variable abundance in both epipelagic and mesopelagic waters and is referred to as “middle OTU”. All other Thaumarchaeota OTUs were grouped into those three OTU types, according to their abundance pattern.

**Figure 3:**
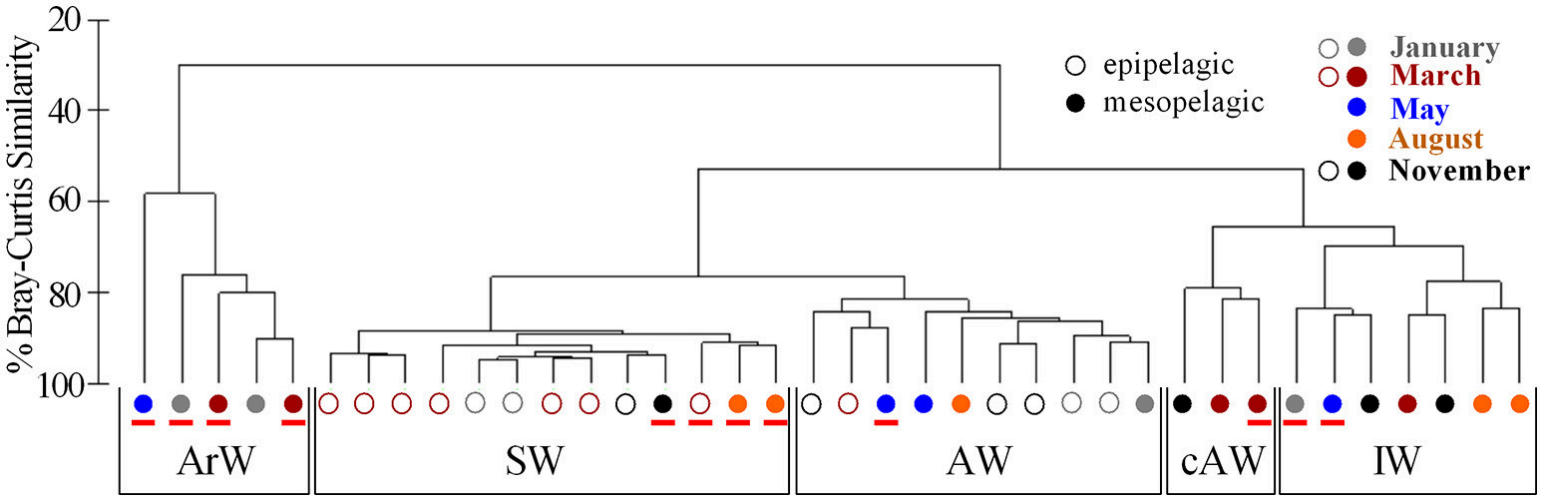
Hierarchical cluster analysis of Bray-Curtis similarities, based on the diversity of Thaumarchaeota sequences from Illumina amplicon sequencing of 16S rRNA genes, illustrating the distribution of samples into five groups. These five groups are labeled according to water mass specificities of the samples within the groups (ArW, Arctic Water; SW, Surface Water; AW, Atlantic Water; cAW, cold Atlantic Water; IW, Intermediate Water). Samples with a mismatch between their associated water mass group and the physical water mass of their sampling origin are marked with a red line. The sampling month is given by colors. Samples were further distinguished according to depth, with open circles for epipelagic samples (0–200 m; *n* = 16) and filled circles for mesopelagic samples (200–1,000 m; *n* = 22).

**Figure 4:**
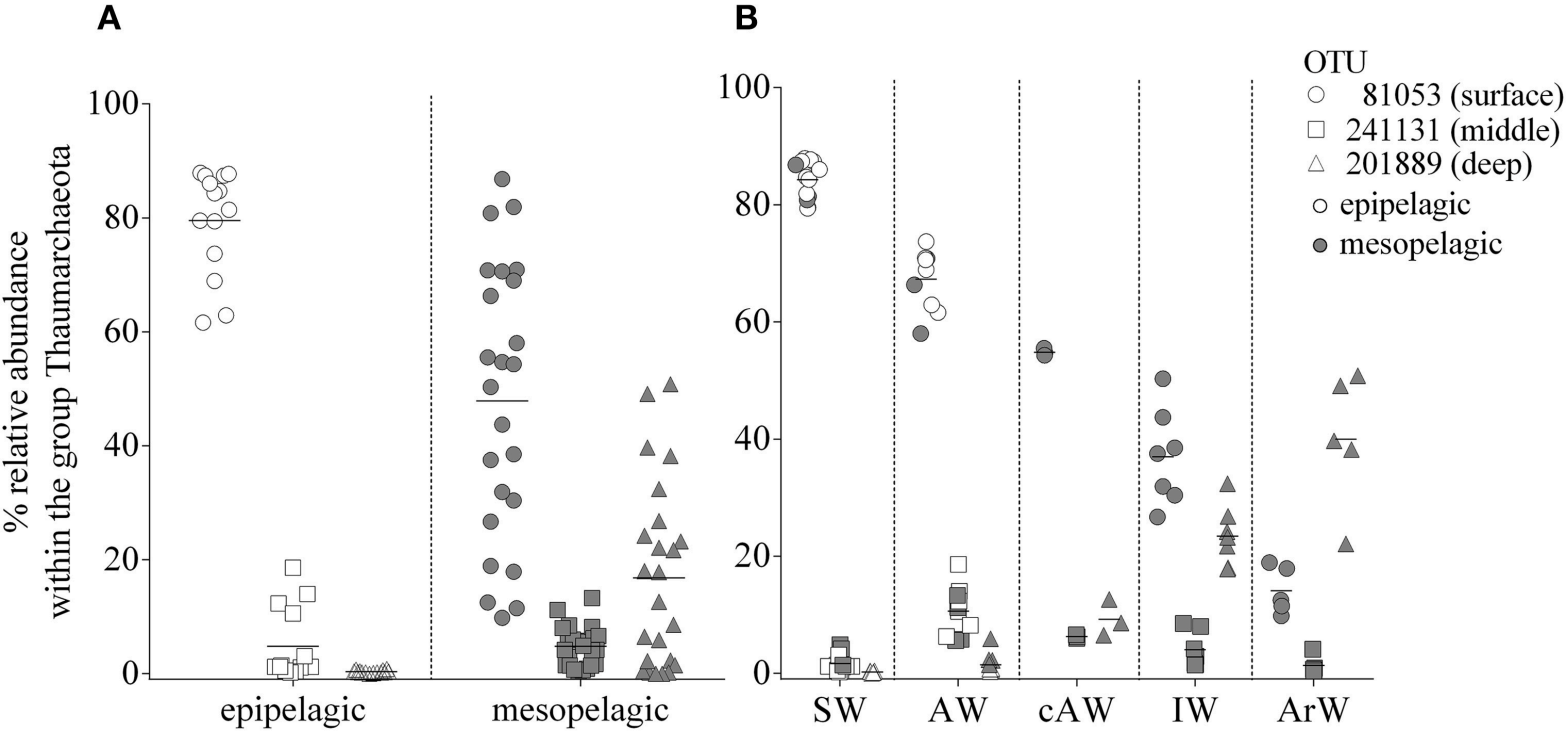
**(A)** Relative abundance of the three most abundant Thaumarchaeota OTUs. Samples were divided according to their sampling origin in epipelagic (open symbols; 0–200 m; *n* = 16) and mesopelagic (filled symbols; 200–1,000 m; *n* = 22) zones. **(B)** Same data as in **(A)**, grouped according to assigned water masses based on sample similarities illustrated in Figure 3. ArW, Arctic Water; SW, Surface Water; AW, Atlantic Water; cAW, cold Atlantic Water; IW, Intermediate Water.

In order to identify Thaumarchaeota distribution patterns throughout the entire sample set, a cluster analysis using Bray-Curtis similarities was performed (Figure 3 and Supplementary Figure S4). The cluster analysis shows five groups, which are in co-occurrence with the five physical water masses observed in the study area (exceptions marked in red). This co-occurrence pattern was observed over the entire sampling period and the Thaumarchaeota abundance pattern in January in AW samples was highly similar to AW samples from March, May August and November (Figure 3). Therefore grouping the samples into the water mass groups revealed a more distinct distribution pattern of the three most abundant OTUs, shown to be significantly different by an ANOSIM analysis (*R* = 0.63; *p* = 0.001) (Figure 4B). The different water mass groups include samples from varying depths and some from both epipelagic and mesopelagic zones, as indicated in Figure 4B.

The influence between water mass and Thaumarchaeota OTU abundance was particularly noticeable at the two stations in January (Figures 2A,C). Only 43 km apart, the two stations showed very different Thaumarchaeota abundance patterns at comparable depths, connected to the discriminating water masses observed at the stations. These OTU abundance patterns seen in January were also observed at other stations during other sampling months. Over the entire sampling period, the three OTUs showed distinctive changes in abundance in the five water masses. The surface OTU showed highest abundance in SW (84% ± 3.1) and declined in the other water masses down to 14% ± 3.6 in ArW. An opposite trend was observed for the deep OTU with highest abundance in ArW (40%±10) declining in IW (23% ± 4.7), cAW (9.2% ± 2.5), AW (1.5% ± 1.7), and lowest in SW (0.2% ± 0.2). The highest abundance of the middle OTU was found in AW (11% ± 4.0) and declining in the other modified Atlantic water masses (cAW: 6.3% ± 0.2; IW: 4.0% ± 2.8). In the SW and ArW the middle OTU was underrepresented (1.7% ± 1.4; 1.3% ± 1.4).

We tested whether the changes in relative abundance of the three OTUs were significantly correlated with the different water masses and other environmental factors. The best fit for linear regressions was achieved when Thaumarchaeota abundance was plotted against water mass and not depth. All three OTUs showed distinct seasonally reoccurring abundance patterns that correlate with the distinct water masses in the area. OTU abundance either decreased (surface and middle OTU, *R*^2^ = 0.96; *p* < 0.0001 and *R*^2^ = 0.6; *p* < 0.0001) or increased (deep OTU, *R*^2^ = 0.84; *p* < 0.0001) from SW or AW toward deeper water masses, such as cAW, IW, and ArW (Figure 4B). Environmental factors were correlated with some OTUs but not all three together. For example, both the middle OTU and surface OTU were positively (Pearson's r, *r* = 0.51; *p* < 0.0014) or negatively (Pearson's r, *r* = −0.51; *p* < 0.0012) correlated respectively to salinity, whilst the deep OTU was not correlated at all. Temperature was also correlated with abundance of the middle OTU (Pearson's r, *r* = 0.69; *p* < 0.0001), but not the other OTUs. None of the three OTUs was correlated with ammonium concentration or sampling month.

### Analysis of amoA gene phylogeny

**Figure 5:**
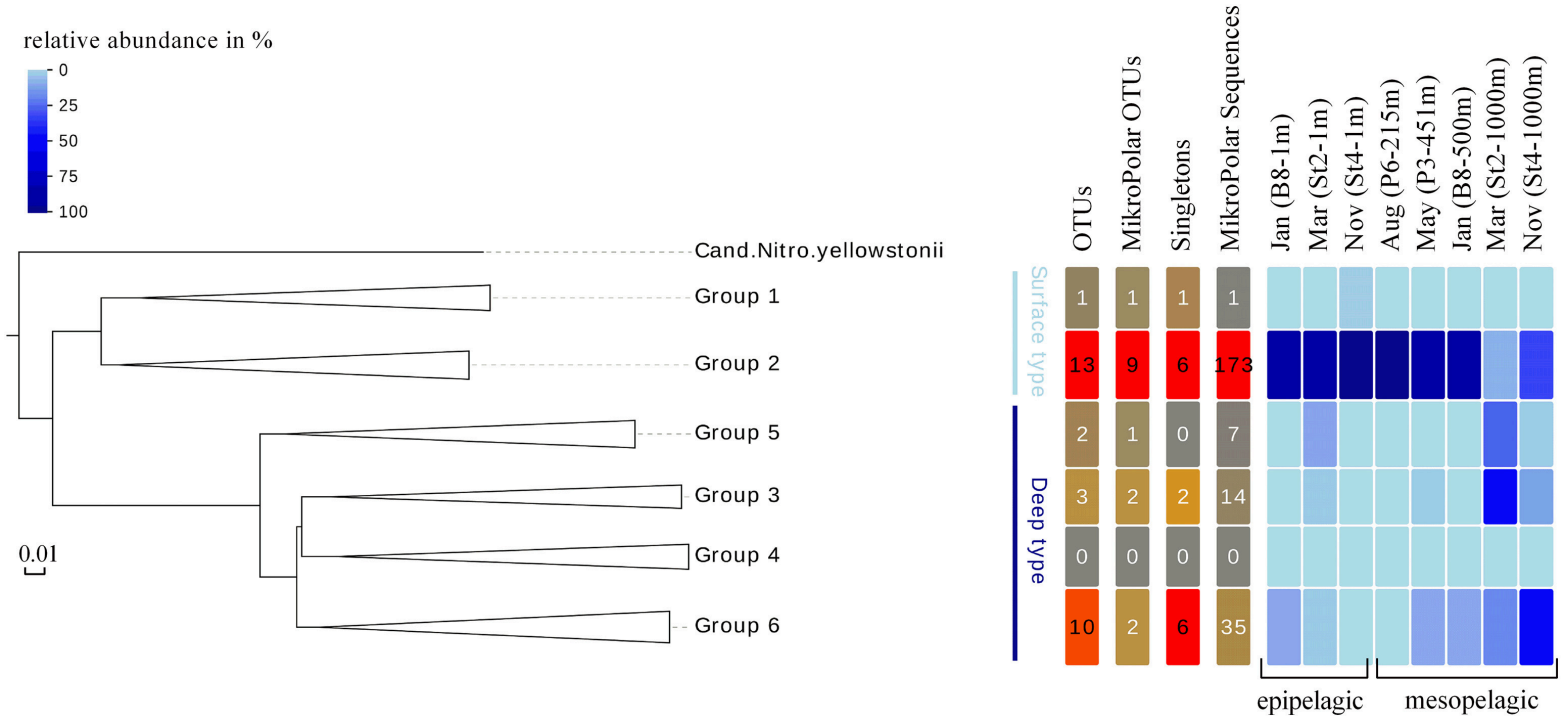
**Left panel** Phylogenetic tree of 189 *amoA* OTUs at 97% sequence identity, representative of the genetic diversity of *amoA* clades 1-6 in reference samples (254 sequences) and in 10 MicroPolar samples (230 sequences). The colored lines indicates the grouping of the six *amoA* clusters in surface (light blue) and deep (dark blue) type. **Right panel** Distribution of OTUs, OTUs which only consist of MicroPolar sequences, OTUs that only consist of one sequence and MicroPolar sequences within the six *amoA* clusters. Heat map, in shades of blue, displaying the relative abundance of OTUs at each analyzed station and grouped by sampling depth.

**Figure 6:**
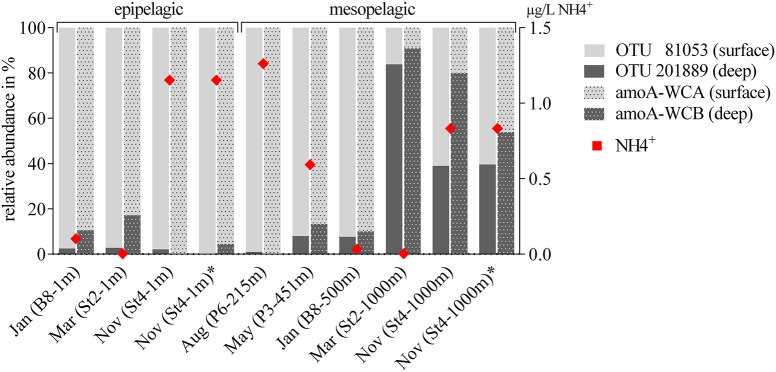
Comparison of the relative abundance of Thaumarchaeota OTUs (16S rRNA gene) and the functional gene for ammonia oxidation (*amoA*). Thaumarchaeota OTUs are divided into the two most abundant sequences (OTU 81053 and OTU 201889), with highest relative abundance at the surface or the deep, respectively. *amoA* OTUs were divided into the two depth depending clusters WCA (surface) and WCB (deep). One station for each sampling month with a surface and deep sampling point were chosen (note, due to very low abundances, summer surface samples are not included), including all five water masses encountered during the five cruises. Ammonium concentrations at each sampling point are visualized as red diamonds (0 = below the detection limit).

To answer whether there was a similar Thaumarchaeota distribution pattern on the functional gene level, we analyzed the distribution of the gene encoding for amoA. A single station for each sampling month (comprising both a surface and deep sampling point) was chosen, excluding summer season surface samples with the low Thaumarchaeota abundance. These eight samples represented all different water masses encountered during the five cruises and resulted in 230 *amoA* sequences. The genetic diversity of these MicroPolar sequences, combined with published sequences from the entire Atlantic, is illustrated in Supplementary Figure S2 and revealed the six main subclusters previously reported (Sintes et al., 2016). A simplified version of this phylogenetic tree is shown in Figure 5. MicroPolar sequences can be found both in subclusters 1 and 2 (representing sequences from WCA) and 3–6 (excluding 4; representing sequences from WCB). In total 15 (5 with >1 sequence) new *amoA* OTUs representing 50 MicroPolar sequences were identified. The majority of our Arctic *amoA* sequences affiliated to subclusters 2 and 6. The deep subcluster 6 represented sequences mostly from mesopelagic samples, while sequences from subcluster 2 were from both epipelagic and mesopelagic MicroPolar samples. The heat map (illustrating the relative abundance pattern both of the WCA and WCB *amoA* sequences) shows only two samples (Mar-St2-1,000 m and Nov-St4-1,000 m) where *amoA* OTUs were more abundant in the deep WCB group than the surface WCA group (Figure 5). Other mesopelagic samples from January, May and August comprised mainly sequences from the WCA group. We compared the 10 samples from the *amoA* data set with our 16S rRNA gene Illumina amplicon data as we observed a similar pattern between the deep and surface OTU types (Figure 6). The different ratio of contrasting 16S rRNA gene surface/deep types was highly similar to *amoA* gene WCA/WCB types in all samples. For both genes the observed pattern was co-occurring with different water masses.

## Discussion

Thaumarchaeota are ubiquitous in marine environments, but a temporal pattern, where abundances decrease significantly during summer seasons, has been described for the polar regions (Massana et al., 1998; Murray et al., 1998; Church et al., 2003; Alonso-Sáez et al., 2008; Christman et al., 2011). We detected high Thaumarchaeota abundances in winter surface waters, contributing up to 38% to the total prokaryotic community. High Thaumarchaeota abundance in surface waters have been reported before, with maximum values of 64% in the Antarctic (Kalanetra et al., 2009) or up to 40% in the Northern Gulf of Mexico (Tolar et al., 2013). Measurements of absolute Thaumarchaeota abundance in other parts of the Arctic Ocean (Amundsen Gulf region) showed similar high gene copy numbers (10^5^ 16S rRNA gene copies mL^−1^) in winter surface water as observed in our study. Our data confirmed a cyclical shift in Thaumarchaeota abundance in surface waters, showing a strong increase in winter months and a decline in summer months (Figure 1B). At the taxonomic level of OTUs, we observed a distinct distribution of different Thaumarchaeota ecotypes that were not directly correlated with their epipelagic or mesopelagic sampling origin. These ecotypes rather seemed to occur according to different water masses, representing a possibly important (yet often neglected) environmental factor as the main driver of Thaumarchaeota distribution. This study better defines how water masses may influence the abundance of Thaumarchaeota in the ocean. Water masses combine by definition a set of measurable environmental parameters, extending the three, including salinity, density and temperature used to define them, as well as factors like origin and history. Due to this complexity it remains unclear which environmental parameter is driving the distribution of Thaumarchaeota OTUs. However, the results of this study point toward a more complex mechanism of Thaumarchaeota distribution than, as previously reported, depth or ammonium concentration could explain.

### Thaumarchaeota abundance patterns correlated with specific water masses

The 16S rRNA gene sequence data showed that the surface Thaumarchaeota group is dominated by a single OTU, which has previously been found both in the Arctic and Antarctic Oceans (Arctic 70%: Alonso-Sáez et al., 2012; Antarctic 83%: Kalanetra et al., 2009; Grzymski et al., 2012). The most abundant OTU in our data set was predominantly identified in surface samples, comprising up to 88% of the Thaumarchaeota population and sharing 98% identity with the same OTU from the Arctic and Antarctic studies. As a result of the repeated sampling campaigns of depth profiles over the entire polar year, we detected the reoccurrence of this OTU in the winter surface waters and decreasing abundance with depth. Interestingly, another single OTU outcompeted the surface OTU and was dominant in our mesopelagic samples. However, depth alone could not explain the abundance pattern. Our data indicates a clear distribution and hence niche diversification of Thaumarchaeota ecotypes according to discriminating water masses in this area (Figure 4). A distinct biogeography for Thaumarchaeota in the ocean has been described before, but abundance patterns of different Thaumarchaeota groups were only connected to depth-specific water profile characteristics (Francis et al., 2005; Beman et al., 2008; Sintes et al., 2013, 2016). However, there have been studies where shifts in marine microbial community composition were correlated to differences in physicochemical water mass parameters (Agogué et al., 2008; Galand et al., 2009; Baltar et al., 2016).

For the first time, we have made a causal link between the abundance patterns of different Thaumarchaeota ecotypes to water masses entering the Arctic Ocean. Thaumarchaeota OTUs group primarily into three clusters, with each group having one OTU being most abundant. These three OTUs, putatively defined as surface, middle and deep OTUs, seemingly exhibit a certain niche specificity, as they vary in abundance best explained by water mass distribution and not, as otherwise suggested, depth or ammonium concentration. By applying this principle to our data, twelve out of thirty-eight samples were revised with regard to their physicochemical water mass definition. These revisions however, can be used to explain the hydrographical system in our study area in a more concise way.

For example, according to the physicochemical water mass information, 1,000 m samples taken from stations in the Nansen Basin were different, either assigned to ArW or IW. However, the Thaumarchaeota abundance pattern was highly similar suggesting that they all originated from ArW, which is different from other deep water masses. Based on the molecular data, we therefore conclude that the water mass at 1,000 m throughout the Nansen Basin is ArW. We did not see this Thaumarchaeota pattern in all of our 1,000 m samples, but only from the stations closest to the deep Nansen Basin, indicating that this OTU was not depth-specific, but rather water mass-specific. Additionally, the absence of the surface OTU is indicative that this water mass did not result from mixing of SW or incoming AW, but rather originated from the deep central Arctic Ocean. Another, co-occurrence between an OTU abundance pattern and water mass was found for AW. By following the changes in abundance of the middle OTU (which correlated with higher salinity and warmer sea temperatures) in particular, we could trace the inflow and modification of AW. The surface OTU on the other hand had highest abundances in SW samples, which is influenced by ice melt. This suggests that the microbial assemblage can provide information on the origin of the water masses, in addition to the physical parameters. It further highlights the possibility that water mass definition can go beyond pure physical parameters and by including molecular microbiological data, such as OTU distribution patterns, presented in this study, explain better the origin and development of water masses (Fuhrman and Steele, 2008; Galand et al., 2009; Djurhuus et al., 2017).

The differences in Thaumarchaeota OTU abundance also reveal that water masses act as clearly separated boundaries for the distribution of marine prokaryotes, while we also defined water masses which seemed to be the result of mixing or dilution processes. On the one hand water masses can be considered barriers to microbial dispersal and on the other hand influencing community composition by physical processes like mixing (Agogué et al., 2011; Acha et al., 2015; Djurhuus et al., 2017). This was especially apparent in January, where two stations, just 43 km apart, showed a totally different abundance pattern for the three defined Thaumarchaeota ecotypes throughout the depth profile down to 1,000 m, while overall Thaumarchaeota abundance was comparable at both stations (Figures 2A,C). This is similar to a phenomenon observed at the Subtropical Frontal zone, where community composition of surface water samples was highly different for samples taken only 7 km apart at an oceanic front (Baltar et al., 2016).

Oceanic frontal zones and ocean currents have been considered to be barriers of dispersal (Srivastava and Kratina, 2013). This affects to a high degree the biogeography of microbial communities and is adding a new element in contrast to the idea of strong regional environmental factors structuring the marine communities (Carr et al., 2003). Our data suggests a dual role of water masses in shaping Thaumarchaeota community composition. They both limit and facilitate dispersal of Thaumarchaeota OTUs, which dominate in specific water masses and are distributed when water mass are mixed.

### 16s rRNA and amoA gene phylogeny and global relevance

In our study, the deep OTU was highly abundant in samples up to 500 m, but not in shallower depths (Figure 4). The 16S rRNA gene sequence was the most abundant sequence (68%) of samples taken below 100 m, in a global dataset of 1256 marine Thaumarchaeota sequences (Supplementary Figure S1). The deep OTU sequence was not found in any samples collected above 100 m, whilst the middle OTU (phylogenetically in the same cluster as the deep OTU) was found both in epipelagic and mesopelagic samples. The surface OTU was found in highest abundance in the Arctic surface water in our data set. This same OTU has been recorded in high abundance in surface waters globally and seems to be universally successful under different conditions, having been found both at the Equator and in the Arctic. In fact, none of the three most abundant OTUs in our data set was Arctic-specific and all have been found in marine waters around the globe. Whether those three OTUs are indeed universally successful remains unclear, as the 16S rRNA gene with an OTU definition of 97% similarity might not be suitable to reveal functional ecotype variation.

The distribution of different *amoA* genes was investigated to see if we could identify a similar water mass-dependent pattern as for the 16S rRNA gene data. The relative distribution of the two AOA groups, WCA (surface) and WCB (deep), was found to correspond with the distribution of surface and deep OTUs based on 16S rRNA gene data. By using independent PCR approaches it is however not possible to directly associate the 16S rRNA and *amoA* genotypes, but their observed grouping, co-occurring with different water masses may indicate that the ecotypes defined by the 16S rRNA gene sequence could be functionally different. One hypothesis for this functional difference is the presence of urease genes (*ureC*) in the deep WCB clusters (Swan et al., 2011; Alonso-Sáez et al., 2012; Qin et al., 2014; Tolar et al., 2016). We did not measure the abundance of *ureC* genes in our samples, but it has been shown that Thaumarchaeota ecotypes from Arctic deep waters have a higher abundance of the *ureC* gene than surface groups (Alonso-Sáez et al., 2012). The genomic differences we observe between the surface and deep *amoA* types might be an indicator for evolutionarily different physiological strategies, including the utilization of urea by the deep WCB (Figure 5).

Identifying environmental drivers, which might explain the proportional abundances seen in this study as well as several other studies, will ultimately help understand the ecological role of the different AOA types. Our data indicated a distribution which corresponds to water masses rather than strict depth dependencies. We measured ammonium concentrations at the sampled stations and did not see a correlation between ammonium availability and WCA to WCB (HAC- to LAC-*amoA*) ratio, despite previous reports (Kirchman et al., 2007; Christman et al., 2011; Sintes et al., 2013, 2015, 2016; Santoro et al., 2017). Environmental parameters such as salinity (Francis et al., 2005; Abell et al., 2010), nitrite (Herfort et al., 2007), dissolved oxygen (Santoro et al., 2008), light (Mincer et al., 2007; Merbt et al., 2012), reactive oxygen species (Tolar et al., 2016), and temperature (Biller et al., 2012) have been suggested to regulate Thaumarchaeota community composition. It was further speculated that depth (Biller et al., 2012; Sintes et al., 2013, 2015), which is often correlated with Thaumarchaeota distribution, is a collection of other environmental factors following a gradient (Santoro et al., 2017). We expand that idea by highlighting that water masses, being by definition a set of several environmental parameters, are important for the distribution of Thaumarchaeota OTUs. Ultimately, it is therefore a challenge to comprehensively identify a single primary driver of AOA distribution.

## Conclusion

We observed a co-occurrence of the three dominant Thaumarchaeota OTUs with water masses at the inflow to the Arctic Ocean. This supports the theory that water mass history to a great extent defines the mesopelagic microbial community structure (Galand et al., 2009; Reinthaler et al., 2010). The Thaumarchaeota pattern we observed was possibly a combination of several factors; water mass characteristics seemed to be a significant factor, influencing the distribution of the three most abundant OTUs; additionally, physical mixing or dilution of water masses might be another important factor explaining the differences in abundance of the three Thaumarchaeota ecotypes. Our study highlights the importance of water masses in influencing Thaumarchaeota population distributions. As water mass distributions will change in a future Arctic Ocean, due to processes such as increased sea ice melting (Comeau et al., 2011) or “Atlantification” (Polyakov et al., 2005; Holland et al., 2006; Walczowski and Piechura, 2006), so will the Thaumarchaeota distribution change. Further research is needed to investigate possible ecological implications of such scenarios.

## Author contributions

LØ and GB: led the planning of the study; OM, HA, AR, and LØ: collected and processed samples; In addition, BW: assisted on designing bioinformatic analysis strategies and helped improving the language and grammar of the manuscript; MP: performed flow cytometric analysis; OM, BW, AR and LØ: analyzed data, OM prepared figures and tables and wrote the paper; All authors contributed to discussion and interpretation of the data and writing the paper.

### Conflict of interest statement

The authors declare that the research was conducted in the absence of any commercial or financial relationships that could be construed as a potential conflict of interest.
