# Supplementary material for: Spatiotemporal Dynamics of Ammonia-Oxidizing Thaumarchaeota in Distinct Arctic Water Masses
Source: Front Microbiol. 2018 Jan 23;9:24. doi: 10.3389/fmicb.2018.00024 (PMC5787140; doi:10.3389/fmicb.2018.00024)
Supplement: Supplementary file 1 [file DataSheet1.DOCX]

Supplementary Material

**Spatiotemporal dynamics of ammonia-oxidizing Thaumarchaeota in distinct Arctic water masses**

Oliver Müller^1*^, Bryan Wilson^1^, Maria L. Paulsen^1^, Agnieszka Rumińska^1^, Hilde R. Armo^1^, Gunnar Bratbak^1^, Lise Øvreås^1,2^

^1^Department of Microbiology, University of Bergen, 5006 Bergen, Norway; ^2^University Center in Svalbard, UNIS

*Corresponding author: oliver.muller@uib.no

# Supplementary Figures and Tables

**

**

**Supplementary Figure S1:** *Left panel*) Phylogenetic tree of twenty-three 16S rRNA OTUs (268 bp at variable region 4) at 97% sequence identity, representative of the genetic diversity of Thaumarchaeota in reference samples (1256 sequences) and in one MicroPolar sample from November (six sequences). The label background color differentiates cultured species (red) and environmental sequences (light blue). The blue circle indicates the MicroPolar sequences. The dotted line groups the OTUs based on the two Thaumarchaeota depth types.
*Right panel)* Heat map, in shades of grey, displaying the relative abundance of all OTUs grouped into epipelagic and mesopelagic zones.

**
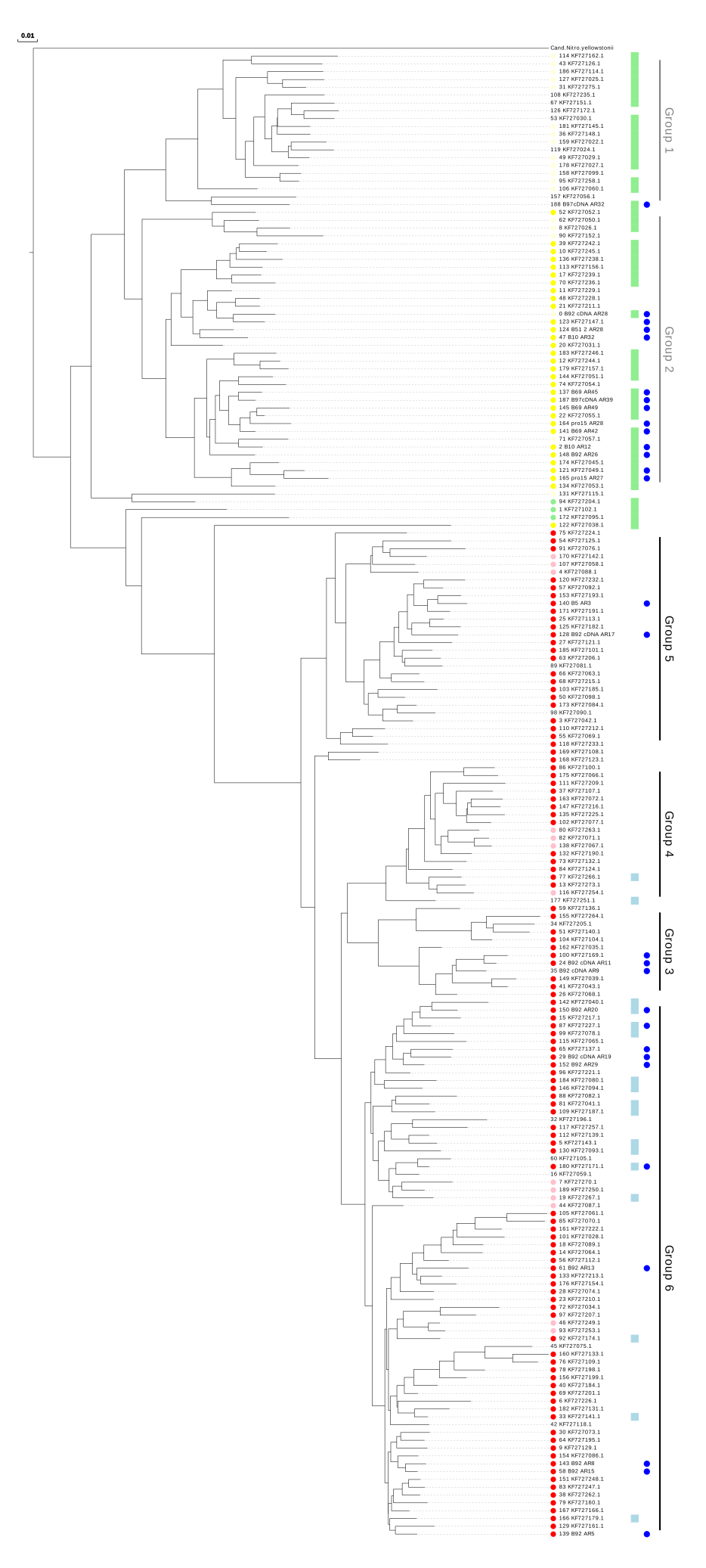
**

**Supplementary Figure S2:** Phylogenetic tree of 190 amoA OTUs at 97% sequence identity, representative of the genetic diversity of *amoA* clades 1-6 in reference samples (254 sequences) and in ten MicroPolar samples (230 sequences). The color codes explain following:
- Circle left of name: yellow (WCA); light yellow (WCA_like); light green (WCA/WCB); red (WCB); light red (WCB_like)
- Rectangle: light green (HAC); light blue (LAC)
- Blue circle: MicroPolar sequences

**

**

**Supplementary Figure S3:** Ratio of Thaumarchaeota relative abundance derived from Illumina amplicon sequencing against qPCR calculated relative abundance (gene copies/mL in relation to flow cytometer counts). Black line shows the linear regression of the data (slope=0.51) and the dotted line indicates a theoretical linear regression with a slope of 1, applicable if relative abundance values from both methods would be equal.

**

**

**Supplementary Figure S4:** Redundancy analysis (RDA) ordination plots of Thaumarchaeota diversity based on sequences from Illumina amplicon sequencing of 16S rRNA genes against A) physical water mass characteristics (explaining 68% of the variation) and B) water mass groups defined by Bray-Curtis similarity of Thaumarchaeota sequences illustrated in Figure 3 (explaining 79% of the variation). The different water masses are indicated by symbols and clusters of samples are colored according to water mass. The sample labels indicate sampling month, depth and station.

| **Supplementary Table S1:** Sampling information and chemical parameters for samples taken during the five cruises around Svalbard in 2014. SW=Surface Water, AW=Atlantic Water, cAW=Cold Atlantic Water, IW=Intermediate Water, ArW=Arctic Water; NA=Not Available | | | | | | | | | | |
| --- | --- | --- | --- | --- | --- | --- | --- | --- | --- | --- |
| Month | Station | Latitude (°North) | Longitude (°East) | Depth | Water Mass (physical) | Water Mass (Thaumarchaeota) | Temperature (°C) | Salinity (µM) | Density (kg m-3) | NH4  (in µg) |
| January | B16 | 81.77 | 19.16 | **1** | SW | SW | -1.85 | 34.28 | 27.60 | 0.49 |
|  |  |  |  | **20** | SW | SW | -1.85 | 34.28 | 27.60 | 0.42 |
|  |  |  |  | **500** | cAW | **ArW** | 0.95 | 34.91 | 27.97 | 0.05 |
|  |  |  |  | **1000** | ArW | ArW | -0.27 | 34.90 | 28.04 | 0.22 |
|  | B8 | 81.43 | 17.88 | **1** | AW | AW | 2.20 | 34.93 | 27.90 | 0.10 |
|  |  |  |  | **20** | AW | AW | 2.19 | 34.93 | 27.90 | 0.08 |
|  |  |  |  | **500** | AW | AW | 2.28 | 35.03 | 27.97 | 0.03 |
|  |  |  |  | **1000** | IW | **cAW** | -0.25 | 34.92 | 28.05 | 0.17 |
| March | St1 | 80.77 | 16.12 | **20** | AW | AW | 3.13 | 35.04 | 27.90 | 0.19 |
|  | St2 | 82.00 | 20.02 | **1** | SW | SW | -1.80 | 34.44 | 27.69 | 0.00 |
|  |  |  |  | **320** | cAW | **IW** | 2.00 | 34.95 | 27.93 | 0.00 |
|  |  |  |  | **1000** | IW | **ArW** | -0.10 | 34.90 | 28.04 | 0.00 |
|  | St3 | 82.52 | 19.39 | **1** | SW | SW | -1.80 | 34.36 | 27.68 | 2.11 |
|  |  |  |  | **20** | SW | SW | -1.50 | 34.40 | 27.68 | 0.08 |
|  | St4 | 82.38 | 19.87 | **1** | SW | SW | -1.78 | 34.40 | 27.71 | 0.09 |
|  |  |  |  | **20** | SW | SW | -1.70 | 34.44 | 27.72 | 0.60 |
|  | St5 | 82.55 | 21.03 | **20** | SW | SW | -1.70 | 34.40 | 27.73 | 0.28 |
|  |  |  |  | **320** | AW | **IW** | 2.10 | 34.90 | 27.93 | 0.10 |
|  |  |  |  | **1000** | IW | **ArW** | -0.45 | 34.55 | 28.04 | 0.00 |
|  | St6 | 80.86 | 15.08 | **20** | AW | AW | 3.20 | 35.03 | 27.89 | 0.00 |
|  |  |  |  | **1000** | IW | IW | -0.40 | 34.91 | 28.06 | 0.00 |
| May | P1 | 79.98 | 10.71 | 1 | SW | NA | 1.01 | 34.52 | 27.66 | 0.07 |
|  |  |  |  | 10 | SW | NA | 1.87 | 34.82 | 27.84 | 0.15 |
|  |  |  |  | **365** | cAW | cAW | 3.00 | 35.13 | 28.03 | 0.05 |
|  | P3 | 79.72 | 9.46 | 1 | SW | NA | -0.34 | 34.07 | 27.37 | 0.17 |
|  |  |  |  | 10 | SW | NA | -0.19 | 34.16 | 27.44 | 0.37 |
|  |  |  |  | **375** | cAW | **AW** | 2.96 | 35.11 | 28.03 | 0.59 |
|  | P4 | 79.77 | 6.27 | 1 | SW | NA | -0.97 | 33.74 | 27.13 | 0.3 |
|  |  |  |  | 10 | SW | NA | -0.97 | 33.83 | 27.70 | 0.24 |
|  |  |  |  | **500** | AW | AW | 2.70 | 35.09 | 27.99 | 0.16 |
|  |  |  |  | **1000** | IW | **ArW** | -0.81 | 34.93 | 28.09 | 0.23 |
| August | P5 | 79.97 | 10.73 | 1 | SW | NA | 6.03 | 34.86 | 27.44 | 0.15 |
|  |  |  |  | 20 | SW | NA | 5.79 | 34.95 | 27.54 | 0.8 |
|  |  |  |  | **200** | AW | **SW** | 4.20 | 35.12 | 27.88 | 1.26 |
|  | P6 | 80.86 | 15.02 | 1 | SW | NA | -1.00 | 31.86 | 25.61 | 1.19 |
|  |  |  |  | 25 | SW | NA | -1.12 | 32.96 | 27.24 | 0.1 |
|  |  |  |  | **500** | AW | AW | 2.16 | 35.04 | 27.99 | 0.44 |
|  |  |  |  | **1000** | IW | IW | -0.46 | 34.93 | 28.07 | 0.00 |
|  | P7 | 80.71 | 15.27 | 1 | SW | NA | 0.11 | 31.69 | 25.43 | 0.02 |
|  |  |  |  | 25 | AW | NA | 4.95 | 34.75 | 27.50 | 0.1 |
|  |  |  |  | **500** | AW | **SW** | 3.40 | 35.10 | 27.93 | 0.00 |
|  |  |  |  | **1000** | IW | IW | -0.24 | 34.94 | 28.07 | 0.16 |
| November | St1 | 78.99 | 10.00 | **20** | SW | SW | 3.30 | 34.83 | 27.72 | 0.00 |
|  | St2 | 79.01 | 1.97 | **750** | cAW | **IW** | 0.50 | 34.94 | 28.03 | 0.00 |
|  | St3 | 79.02 | 11.33 | **20** | SW | SW | 2.30 | 34.59 | 27.62 | 0.54 |
|  |  |  |  | **300** | AW | AW | 3.63 | 35.11 | 27.91 | 1.48 |
|  | St4 | 79.03 | 6.01 | **5** | AW | AW | 3.66 | 35.09 | 27.90 | 1.15 |
|  |  |  |  | **20** | AW | AW | 3.67 | 35.09 | 27.89 | 0.35 |
|  |  |  |  | **500** | cAW | cAW | 1.87 | 35.03 | 28.01 | 0.96 |
|  |  |  |  | **1000** | IW | IW | -0.39 | 34.94 | 28.07 | 0.83 |

| **Supplementary Table S2:** Table of primers used in this study | | | |
| --- | --- | --- | --- |
| Primer | Sequence | Reference | Annealing temp. |
| 519F | CAGCMGCCGCGGTAA | Øvreås et al, 1997 | 55 |
| 806R | GGACTACHVGGGTWTCTAAT | Caporaso et al. 2011 |  |
| Arch-amoAF | STAATGGTCTGGCTTAGACG | Francis et al. 2005 | 53 |
| Arch-amoAR | GCGGCCATCCATCTGTATGT | Francis et al. 2005 |  |
| Arch21F | TTCCGGTTGATCCYGCCGGA | Delong 1992 | 52 |
| Uni1492R | NTACCTTGTTACGACT | Loy et al. 2002 (modified) |  |

**Supplementary Table S3:** Table describing characteristics determining the water masses encountered during the five cruises around Svalbard. For further explanation see (Cokelet, Tervalon, and Bellingham 2008).

| Water Masses | Temperature (T in °C)/ or Density (σ_T_) | Salinity (PSU) |
| --- | --- | --- |
| Atlantic Water | T >2 °C | S >34.9 |
| cold Atlantic Water | 0<T<2 °C | S >34.9 |
| Intermediate Water | T<0 °C | S >34.9 |
| Surface Water | σ <27.7 | S <34.92 |
| Arctic Water | σ >27.7 | S <34.92 |
